# Miliary Lung Lesions Mimicking Tuberculosis: A Case of Metastatic Lung Adenocarcinoma

**DOI:** 10.7759/cureus.88178

**Published:** 2025-07-17

**Authors:** Mahmoud Ali, Mahmoud Helaly, Abdulrahman Irhouma, Shamsaldeen Abdelraheem

**Affiliations:** 1 Intensive Care Unit, North Manchester General Hospital, Manchester, GBR; 2 Critical Care Medicine, Manchester University National Health Service (NHS) Foundation Trust, Manchester, GBR; 3 General Medicine, Manchester University National Health Service (NHS) Foundation Trust, Manchester, GBR

**Keywords:** adenocarcinoma lung, chest lung cancer interstitial lung desase infective desease, disseminated tuberculosis, miliary lesions, respiratory illness

## Abstract

Miliary patterns on chest imaging frequently prompt urgent consideration of disseminated infectious diseases such as tuberculosis (TB), particularly in patients from endemic regions. However, non-infectious aetiologies like metastatic malignancy must also be considered. We report the case of a 39-year-old previously healthy male with miliary pulmonary lesions initially treated empirically for disseminated TB. Despite therapy, his condition deteriorated until biopsy results confirmed metastatic lung adenocarcinoma. This case highlights the diagnostic challenges in TB-endemic regions and underscores the importance of early tissue diagnosis.

## Introduction

Miliary lung patterns are characterised by innumerable small (1-3 mm) nodules distributed diffusely throughout both lungs. Common causes include infectious diseases such as tuberculosis, fungal infections (e.g., histoplasmosis), and granulomatous diseases like sarcoidosis and pneumoconiosis [1,2]. Among these, TB remains the most prevalent due to its global burden and transmissibility [3]. In TB-endemic areas, empirical anti-TB therapy is often initiated based on radiological findings such as bilateral reticulonodular opacities and lymphadenopathy, classic features of miliary TB [4].

However, non-infectious causes like metastatic malignancy, particularly lung adenocarcinoma, can closely mimic TB, posing significant diagnostic challenges [5,6]. These overlaps may lead to diagnostic delays, especially when treatment is based on assumption rather than definitive tissue diagnosis [7].

Lung adenocarcinoma presenting with a miliary pattern is rare, especially in young, non-smoking individuals. Advances in molecular diagnostics and immunohistochemistry have improved diagnostic precision, highlighting the need for early biopsy in atypical presentations [7].

This case illustrates the importance of early tissue sampling and diagnostic flexibility. It highlights the risk of premature closure in TB-endemic settings and calls for caution when empirical treatments fail.

## Case presentation

Patient demographics and history

A 39-year-old male from a TB-endemic country presented with a two-month history of progressive respiratory symptoms, including increasing fatigue, exertional dyspnoea, a dry, persistent cough, and unintentional weight loss of 10 kg over six weeks. He was a non-smoker, with no known TB exposure, no alcohol or drug use, and no significant medical history. He had travelled briefly to Saudi Arabia several weeks prior to symptom onset, where he experienced a short episode of febrile illness and gastrointestinal upset.

Initial examination and investigations

**Figure 1:**
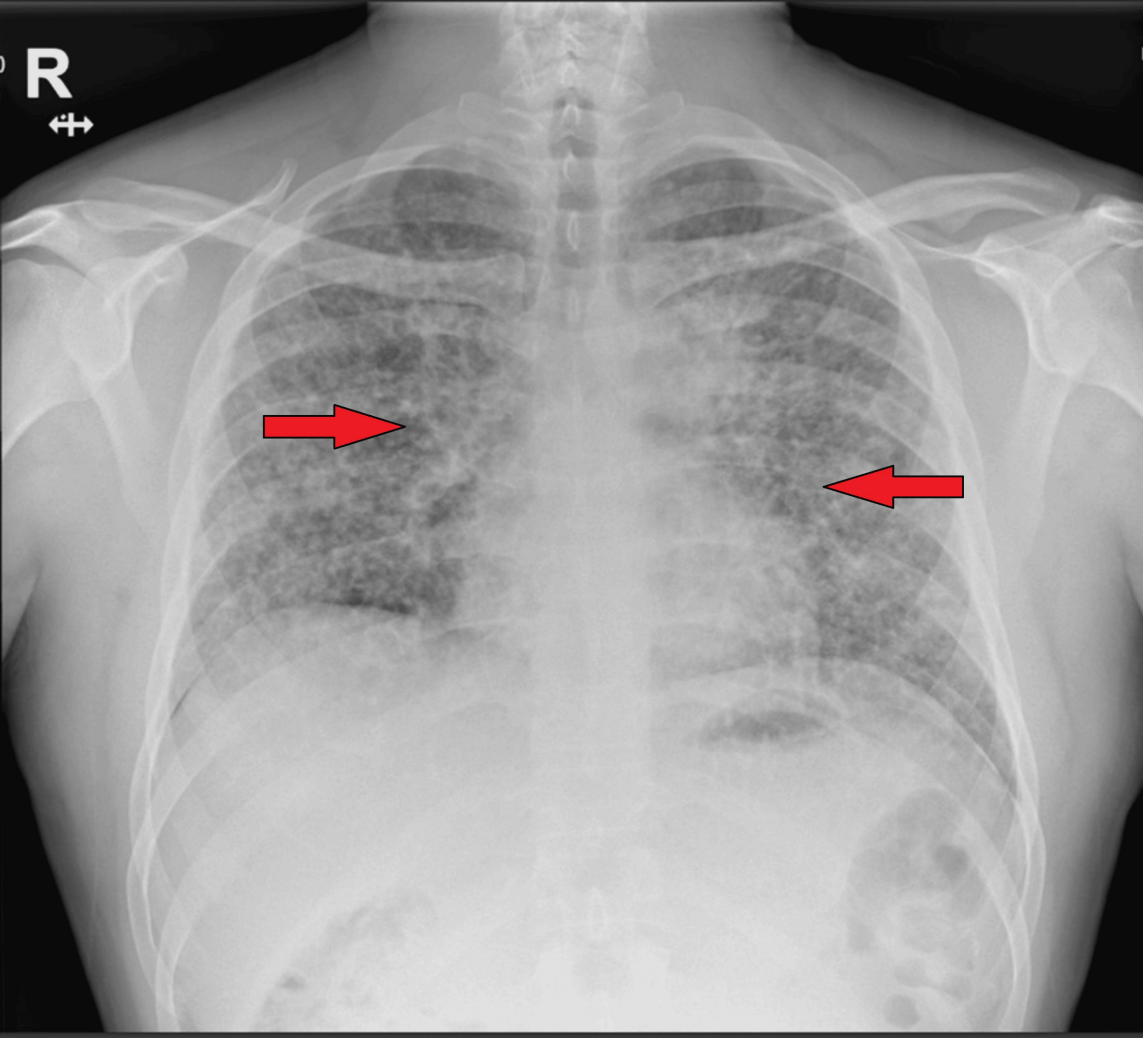
AP chest radiograph showing diffuse bilateral interstitial and reticulonodular opacities. Chest radiograph (anteroposterior [AP] view) showing diffuse bilateral reticulonodular opacities involving mid and lower zones. Lung fields showed increased interstitial markings and ground-glass opacities. No pleural effusion or pneumothorax

On admission, there were no neurological symptoms; he was alert, with a Glasgow Coma Scale (GCS) of 15, no focal deficits, and no seizures or altered mental status. Performance status was Electrocorticography or the Eastern Cooperative Oncology Group (ECOG) 3, indicating limited self-care. The patient appeared cachectic and tachypneic, with an oxygen saturation of 88% on room air. Chest auscultation revealed bilateral basal crackles. A chest X-ray (Figure 1) showed bilateral reticulonodular opacities. High-resolution computed tomography (HRCT) of the chest demonstrated diffuse 1-3 mm miliary nodules with mediastinal lymphadenopathy.

**Figure 2:**
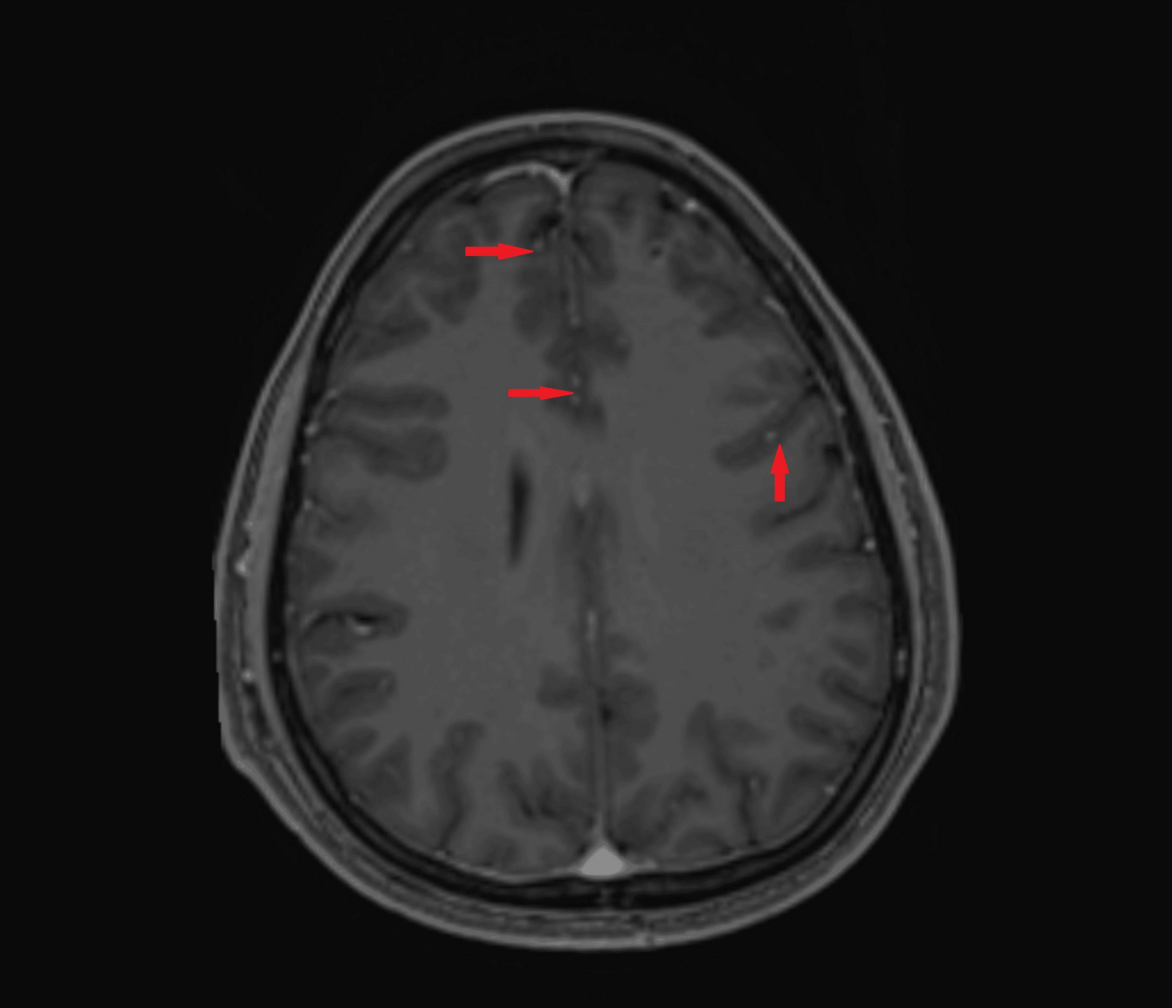
Axial T1-weighted post-contrast MRI of the brain. Multiple tiny supratentorial and infratentorial intra-axial lesions. Most of the lesions are punctate and demonstrate postcontrast enhancement. A few larger lesions in the right frontal lobe white matter and left frontal lobe grey-white matter junction demonstrate peripheral ring enhancement. A few non-enhancing lesions demonstrate blooming artefact in the parietal lobe.

**Figure 3:**
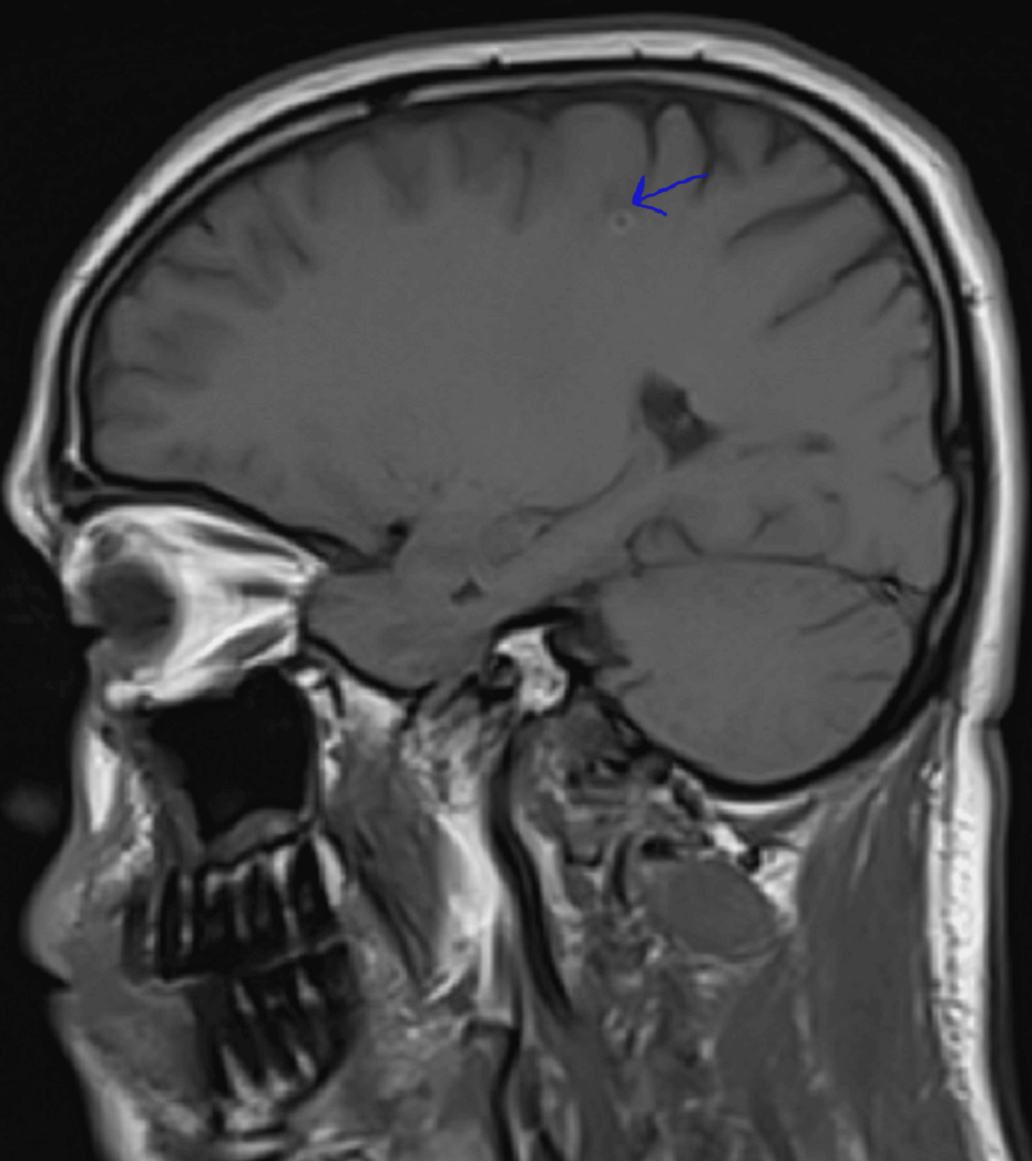
Sagittal T1-weighted post-contrast MRI of the brain. The image shows symmetrical cortical and subcortical structures without evidence of abnormal contrast enhancement. The ventricles are midline and of normal size. No mass effect, midline shift, or abnormal enhancement is observed. Multilobar bilateral supratentorial and infratentorial tiny lesions that demonstrate high T2 signal and high T1 signal. The lesions are predominantly subcortical and at the grey-white matter junction. The largest lesion measures 5 mm with a ring morphology.

An MRI of the brain was performed for lethargy (but no focal neurological signs). Imaging revealed multiple small ring-enhancing supratentorial and infratentorial lesions (Figures 2, 3), suggestive of central nervous system (CNS) metastases. However, the patient had no focal neurological deficits, seizures, or altered mental status.

A lumbar puncture revealed normal opening pressure, with unremarkable cerebrospinal fluid (CSF) analysis and negative mycobacterial polymerase chain reaction (PCR), smear, and cultures. Human immunodeficiency virus (HIV) testing was negative.

Multiple respiratory samples, including sputum, bronchial lavage, and endobronchial ultrasound (EBUS)-guided mediastinal lymph node biopsies, were all negative for acid-fast bacilli, TB-PCR, and cultures.

Empirical treatment initiation

Despite the lack of microbiologic confirmation, empirical anti-TB treatment was initiated due to imaging findings and endemic exposure history. The patient was started on the RIPE regimen (rifampicin, isoniazid, pyrazinamide, and ethambutol) along with corticosteroids for presumed central nervous system tuberculosis (CNS TB).

Clinical deterioration

Despite adherence to therapy, the patient experienced progressive clinical decline. Over the next five weeks, his cough and dyspnoea worsened, and he developed daily post-medication nausea and vomiting. He reported profound fatigue, decreased appetite, and further weight loss. There were no fevers, night sweats, haemoptysis, or neurological deficits.

**Figure 4:**
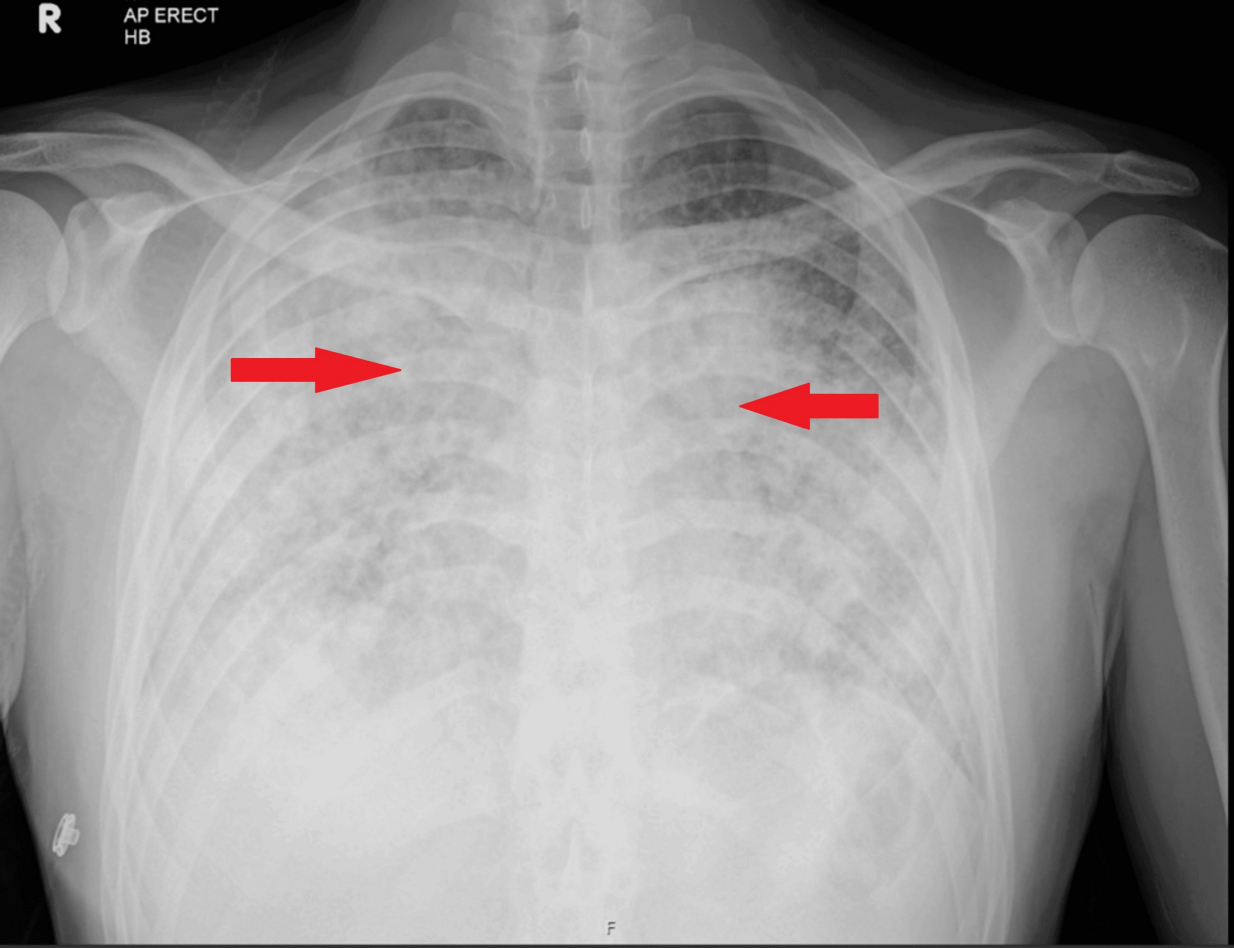
CXR of chest anteroposterior (AP) view. Worsening of bilateral infiltrates compared to previous chest X-ray (CXR).

**Figure 5:**
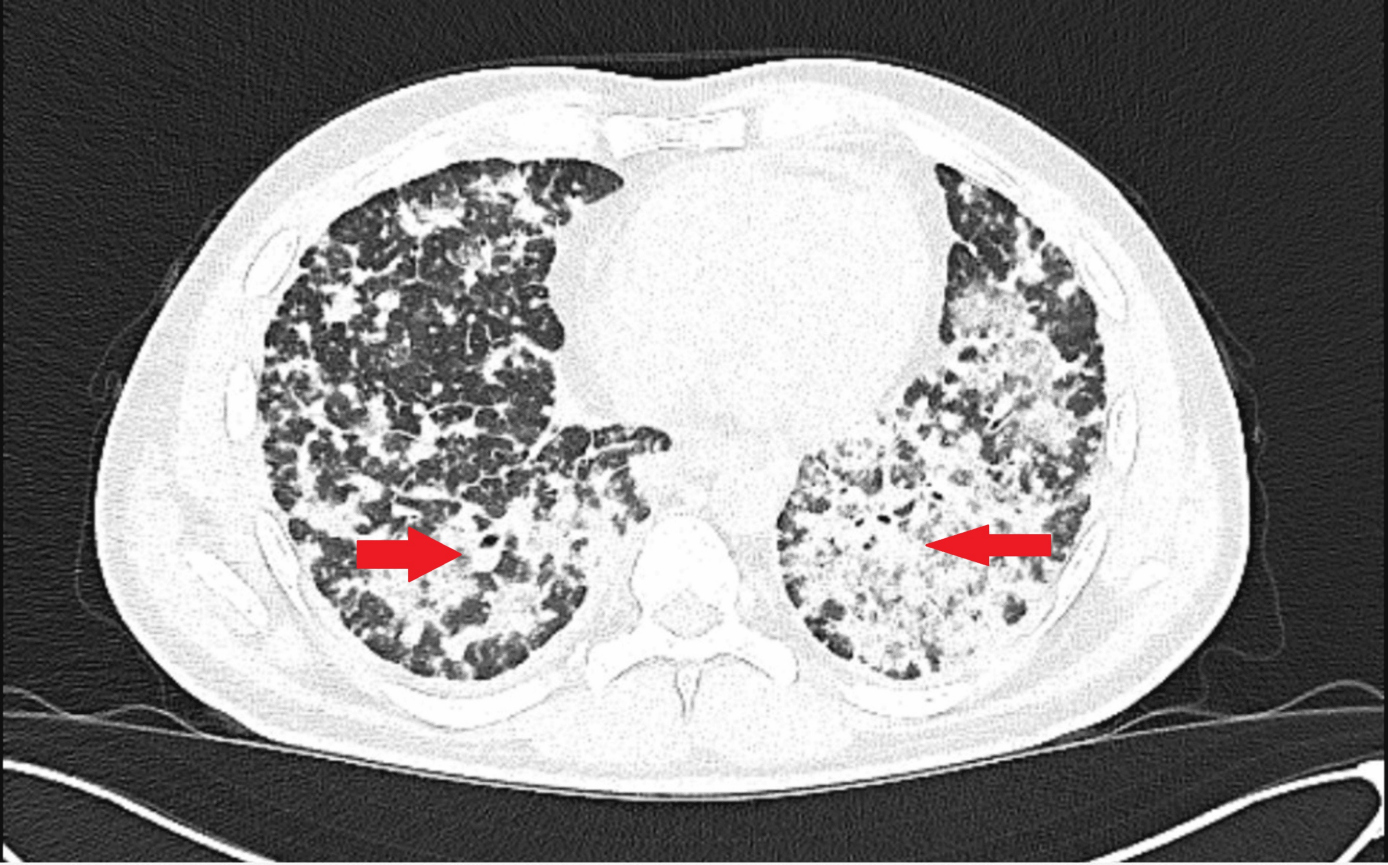
High-resolution CT (HRCT) chest – axial view. This axial High-resolution computed tomography (HRCT) image of the thorax demonstrates extensive bilateral interstitial lung disease with a diffuse pattern of reticular opacities, honeycombing, and ground-glass opacities, predominantly involving the peripheral and basal regions of both lungs.

Repeat imaging showed worsening pulmonary nodules and new lytic bone lesions in the thoracic/lumbar vertebrae and pelvis (Figures 4, 5). These findings raised concern for an alternative diagnosis, particularly metastatic malignancy. A neck ultrasound revealed enlarged supraclavicular lymph nodes, prompting a repeat biopsy.

Histopathological findings

A neck ultrasound identified enlarged supraclavicular lymph nodes.

Histopathological diagnosis

Lymph node biopsy revealed poorly differentiated adenocarcinoma with a solid growth pattern and nucleoli.\n- Immunohistochemistry:

TTF-1 (Thyroid Transcription Factor-1): Positive

CK7 (Cytokeratin 7): Positive

Napsin A: Positive

CDX2 (Caudal Type Homeobox 2): Negative → consistent with primary lung adenocarcinoma\n\n- Molecular Analysis:

ALK and PD-L1: Negative

Next-generation sequencing (NGS) and ROS1 testing were sent, but results were pending at the time of clinical deterioration.

Final clinical course and outcome

Following the diagnosis of metastatic lung adenocarcinoma, the patient’s condition rapidly worsened. He developed respiratory failure requiring high-flow nasal oxygen therapy. Due to his deteriorating performance status and inability to tolerate systemic therapy, he was deemed unsuitable for chemotherapy, immunotherapy, or targeted molecular therapy. 

Palliative care was initiated with symptom-directed management. The patient died shortly thereafter, surrounded by family, following comprehensive communication regarding prognosis and goals of care.

## Discussion

This case illustrates the pitfalls of anchoring bias and premature closure in clinical reasoning, common in TB-endemic settings where miliary TB is often presumed. The presence of CNS lesions and miliary lung nodules supported a diagnosis of disseminated TB. However, the lack of microbiological confirmation and clinical deterioration warranted re-evaluation.

Differential diagnoses initially included sarcoidosis, fungal infections, and metastatic malignancy. Negative TB tests, absence of autoimmune markers, and worsening radiologic findings eventually redirected the clinical suspicion. A delay in tissue diagnosis prolonged the time to definitive management.

TTF-1, CK7, and Napsin A positivity on IHC confirmed lung adenocarcinoma, a disease increasingly seen in non-smokers due to factors such as genetic mutations (e.g., EGFR, ALK) and environmental exposures [5].

Miliary metastases are a rare presentation of lung adenocarcinoma and often portend a poor prognosis. Earlier use of PET-CT or repeat biopsy may have expedited diagnosis. EBUS may miss peripheral or necrotic lesions; thus, core needle or surgical biopsy should be considered if suspicion remains high.

This case underscores the need for early tissue diagnosis and continuous reassessment of empirical treatment, especially when therapy fails or imaging evolves atypically. A takeaway for clinicians: if a patient with suspected TB does not improve within 2-3 weeks and the TB workup is negative, malignancy must be strongly considered.

## Conclusions

In regions where tuberculosis is endemic, a miliary pattern on chest imaging often prompts immediate empirical anti-TB therapy. However, this case demonstrates how relying solely on clinical and radiologic assumptions can delay accurate diagnosis, especially when the true pathology is an atypical presentation of metastatic malignancy. Our patient, a young non-smoker, initially appeared to fit the profile of disseminated TB, yet failed to respond to therapy and was later diagnosed with metastatic lung adenocarcinoma. This highlights the importance of maintaining diagnostic flexibility and pursuing tissue confirmation early in cases of uncertain or deteriorating clinical trajectories.

Lung adenocarcinoma may present with a miliary pattern and affect patients without traditional risk factors, possibly due to genetic predispositions or environmental exposures. Ultimately, when TB microbiological tests are negative and clinical improvement is lacking after 2-3 weeks of treatment, clinicians should reconsider the diagnosis and promptly initiate alternative investigations, including repeat imaging, biopsy, and molecular profiling. Early, confirmatory diagnostics are essential to avoid missed opportunities for timely, potentially life-extending therapy.
